# Microbial Application to Improve Olive Mill Wastewater Phenolic Extracts

**DOI:** 10.3390/molecules26071944

**Published:** 2021-03-30

**Authors:** Flora V. Romeo, Gina Granuzzo, Paola Foti, Gabriele Ballistreri, Cinzia Caggia, Paolo Rapisarda

**Affiliations:** 1 Council for Agricultural Research and Economics (CREA), Research Centre for Olive, Fruit and Citrus Crops, 95024 Acireale, Italy; gina.granuzzo@gmail.com (G.G.); gabriele.ballistreri@crea.gov.it (G.B.); paolo.rapisarda@crea.gov.it (P.R.); 2 Department of Agricultural, Food and Environment, University of Catania, 95123 Catania, Italy; paola.foti@phd.unict.it (P.F.); ccaggia@unict.it (C.C.)

**Keywords:** adsorbent resins, beta-glucosidase, esterase, hydroxytyrosol, *Lactiplantibacillus plantarum*, oleuropein, tyrosol, *Wickerhamomyces anomalus*

## Abstract

Olive mill wastewater (OMW) contains valuable and interesting bioactive compounds, among which is hydroxytyrosol, which is characterized by a remarkable antioxidant activity. Due to the health claims related to olive polyphenols, the aim of this study was to obtain an extract from OMW with an increased level of hydroxytyrosol by means of microbial enzymatic activity. For this purpose, four commercial adsorbent resins were selected and tested. The beta-glucosidase and esterase activity of strains of *Wickerhamomyces anomalus*, *Lactiplantibacillus plantarum,* and *Saccharomyces cerevisiae* were also investigated and compared to those of a commercial enzyme and an *Aspergillus niger* strain. The *W. anomalus* strain showed the best enzymatic performances. The SP207 resin showed the best efficiency in selective recovery of hydroxytyrosol, tyrosol, oleuropein, and total phenols. The bioconversion test of the OMW extract was assessed by using both culture broths and pellets of the tested strains. The results demonstrated that the pellets of *W. anomalus* and *L. plantarum* were the most effective in hydroxytyrosol increasing in phenolic extract. The interesting results suggest the possibility to study new formulations of OMW phenolic extracts with multifunctional microorganisms.

## 1. Introduction

The Mediterranean diet is becoming more and more popular due to its nutritional and health benefits, which are associated with lower incidences of atherosclerosis, certain cancers, and cardiovascular and neurodegenerative diseases [1,2,3,4]. These health benefits can be strongly related to the high content of antioxidant molecules present in food products widely consumed within the Mediterranean diet, such as extra virgin olive oil and table olives. Olives and their industrial derived products are rich in unsaturated fatty acids, tocopherols, and phenols. However, the olive oil industry generates large amounts of high-polluting by-products, such as a solid residue and an effluent commonly named olive mill wastewater (OMW) [5], which is a mix of olive vegetation water and water added during oil extraction, above all with the three-phase centrifugation system [6].

Olive waste still contains most of the valuable and interesting compounds. During the olive oil processing, most of the phenolic compounds are found in the aqueous phase, with a hydroxytyrosol concentration up to 100-fold higher than that found in olive oil [7]. OMW is rich in phenols, such as hydroxytyrosol, tyrosol, oleuropein, flavonoids, and other compounds. These molecules have great potential in the pharmaceutical and nutraceutical fields [8]. In particular, hydroxytyrosol is characterized by remarkable antioxidant activity, which is similar to that of the main synthetic antioxidants [7,9]. In 2011 [10], the scientific opinion on the substantiation of health claims related to olive polyphenols, standardized by their content of hydroxytyrosol and its derivatives (e.g., oleuropein complex), was published by European Food Safety Authority (EFSA).

Substantial interest has been given to the production of hydroxytyrosol both from natural sources and by synthetic procedures [11]. Several techniques have been reported in literature aiming at recovering phenolic compounds from OMW, such as liquid–liquid extraction [12], membrane filtration [13], adsorption on selective polymeric resins [14,15], and enzymatic reactions [11,16,17]. Compared to chemical treatments, enzymatic treatment is advantageous because it requires mild operating conditions, such as pH, temperature, and the absence of toxic organic solvents [18]. To overcome the high costs of enzymes and their poor operational stabilities, new research has focused on enzyme immobilization strategies [19].

Among microorganisms, *Aspergillus* spp. has been extensively studied for its ability to produce extracellular enzymes that can be easily applied to food processing, because they are recognized as Generally Regarded as Safe (GRAS) by the Food and Drug Administration (FDA) [20]. Within the *Aspergillus* genus, *A. niger* is the most efficient producer of β-glucosidase [21]. However, few reports focused on the effect of fungal enzyme addition on OMW phenols have been published, but even less on the direct application of live microorganisms into an OMW extract to improve antioxidant activity. In particular, yeasts and lactic acid bacteria (LAB) appear particularly interesting as they are commonly used as starter cultures in fermented food and widely isolated by OMW or spontaneous fermentation of table olives. During the last two decades, several *Lactiplantibacillus plantarum* strains have been extensively described for their ability to degrade oleuropein and for their probiotic potential [22,23]. Aponte et al. [24] for the first time tested the in vivo bioconversion of oleuropein into hydroxytyrosol by oral granules containing probiotic *L. plantarum* and an olive leaves standardized extract. Their results showed that co-administration of live *L. plantarum* bacteria with the extract provides for higher amounts of bioavailable hydroxytyrosol, compared to the extract alone. Several yeasts isolated from table olives have been found able to produce noticeable enzymes, such as β-glucosidase, and to exhibit probiotic traits [25,26]. Among yeasts, *Saccharomyces cerevisiae* and *Wickerhamomyces anomalus* have been described to produce enzymes useful in the wine making process and their application has been extensively investigated. In addition, *W. anomalus* (formerly known as *Pichia anomala* or *Hansenula anomala*) shows several probiotic traits including cholesterol removal capability [27], and it is an interesting source of different enzymes and a biotechnologically relevant microorganism [28].

In light of this knowledge, this study aimed to investigate the use of live microorganisms together with OMW extracts. In fact, a selection of different types of resins was performed to obtain a phenol-rich extract with the main aim of evaluating the bioconversion activity of different microbial species. The hydroxytyrosol increase in the OMW extract provided by cultures already known as starters is the first step to design innovative formulations of new nutraceuticals supplemented with probiotics.

## 2. Results

### 2.1. Extraction of Phenols from OMW

#### 2.1.1. OMW Analyses

Raw OMW was centrifuged and filtered to remove solid soil residues, olive oil and fragments of olive fruit and stone. The obtained sample was analyzed for the values of pH, dry matter, and total phenols, while hydroxytyrosol (HT), tyrosol (TY), and oleuropein (OLE) concentrations were detected by high performance liquid chromatography (HPLC) (Table 1). These results confirmed the acidic trait of the sample, which showed 6.24 g/100 mL of dry matter value, although subjected to centrifugation, and a high content of total phenolic compounds, for which it is considered a polluting effluent. As expected, among the three quantified peaks, HT showed the highest concentration.

**Figure 1:**
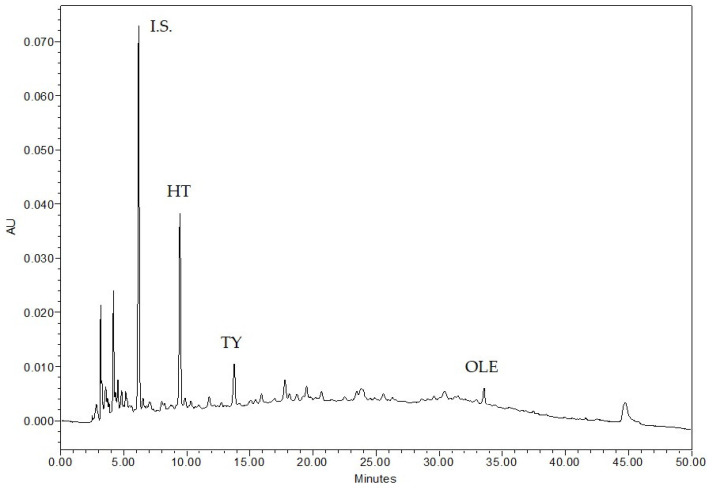
HPLC phenolic profile (at 280 nm) of OMW sample (I.S. = Internal Standard).

Moreover, HT was the most abundant phenol in OMW, as shown in Figure 1. OLE was quantified as gallic acid too, but this was done by calculating the response factor of the instrument in respect to the Internal Standard (I.S.) under the same HPLC operating conditions. HT, TY, and OLE were analyzed as principal markers of OLE enzymatic degradation. They were identified by comparing their retention times and absorption spectra with those of pure commercial standards.

#### 2.1.2. Resins Adsorption

The results of OMW adsorption on resins are shown in Table 2. The results confirmed a different adsorbing resins’ capacity for both polyphenols and individual bioactive compounds. The four tested resins can be divided into three groups. The most efficient were the S-DVB copolymers resins (namely SP207 and XAD16), which adsorbed the highest concentration of total phenolic compounds, followed by the C18 and the PAD900C resins, respectively. Concerning the capacity to adsorb the individual bioactive compounds, the SP207 resin showed the highest efficiency in concentrating HT, TY, and OLE from OMW, whereas the three others (XAD16, PAD900C, and C18 resins) showed a lower efficiency. The extract obtained by the most effective resin (SP207) was then selected for bioconversion experiments.

### 2.2. Enzymatic Tests

The enzymatic activity of three strains was compared to that obtained by the commercial enzyme (Lallzyme beta), and to the activity of germinated conidia of *A. niger* strain. The *A. niger* DSM 2466 was selected among other commercial strains for its proven enzymatic activity (data not shown) and used as positive control. As shown in Table 3, after germination in culture broth, the strain produced the highest value (IU/mL) of both beta-glucosidase and esterase activities. The *A. niger* activity was also higher than that highlighted by the commercial tested enzyme, which is widely used in oenological applications for its known poly-enzymatic activity. Among *W. anomalus, L. plantarum,* and *S. cerevisiae* tested strains, *W. anomalus* was found as the most effective for beta-glucosidase, together with the commercial enzyme and followed by *L. plantarum,* while, for esterase activity, results obtained from *W. anomalus* and *S. cerevisiae* were not statistically different.

### 2.3. Bioconversion Efficiency by W. anomalus and L. plantarum

**Figure 2:**
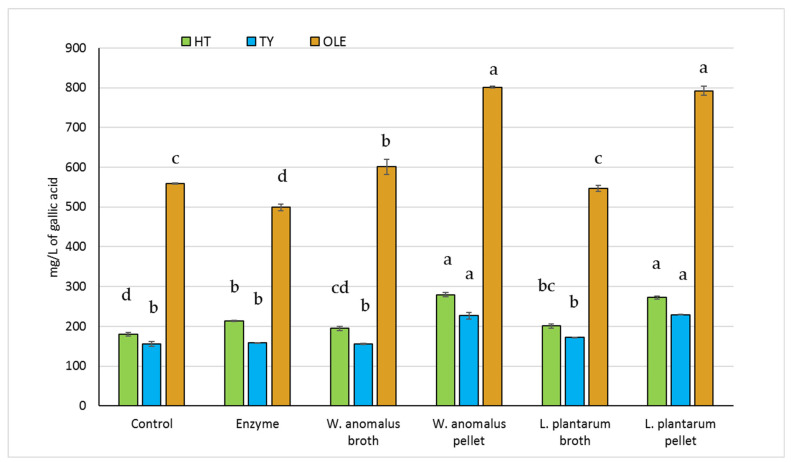
Concentration of hydroxytyrosol, tyrosol, and oleuropein after bioconversion of diluted OMW extract by Lallzyme, extracellular broths and pellets of the two tested strains after 2 h at 50 °C. Different letters indicate statistical differences among the columns of the same compound (Significance at *p* < 0.001).

In the bioconversion test, the two microorganisms showing the best performances at the enzymatic tests (Table 3) were used to evaluate their ability to increase the HT and TY concentrations in the OMW extract. The SP207 extract was used as substrate for both pellets and filtered supernatants. The kinetic analysis of bioconversion was done through HPLC quantification of single phenols up to 6 h. The treatment with the commercial enzyme highlighted a slow and constant increasing of both HT and TY, whereas the treatment with the two tested microorganisms showed the highest value of phenols, reaching the plateau after 2 h of reaction. The only exception was detected after 6 h in the sample treated with pellets, when a further increase of HT value was registered (Table S1, on-line Supplementary Materials). Overall, the highest phenolic values were obtained using the microbial pellets rather than the filtered broths. The mean values of HT, TY, and OLE obtained after 2 h are shown in Figure 2. The highest values of HT were reached using *W. anomalus* and *L. plantarum* pellets (+35.5% and +33.7%, respectively), whereas no statistical difference between the control sample and the *W. anomalus* broth was observed. The TY content increased significantly only after contact with both pellets (+32%). Regarding the OLE amount, although a decrease was registered after 2 h by using the commercial enzyme, its concentration remained almost constant up to 6 h (Table S1). On the contrary, the OLE concentration increased in the presence of both strains’ pellet (+30.2% and +29.5%) and to a lesser degree with *W. anomalus* broth (+7%).

## 3. Discussion

Crude OMW was sampled from a collection tank during a continuous oil extraction from different olive cultivars. The sample showed a pH value of 4.8, within the range of 2.2–5.9 as reported by Dermeche et al. [5], and close to those reported by Aggoun et al. [6] and Dammak et al. [18] who found values between 4.5 and 5.16 for OMW from the three-phase extracting system. Here, the dry matter value was slightly lower than those reported by the previous authors, as a consequence of sample pre-treatment, namely centrifugation and filtration. The total phenols were also lower than those discussed by Belaqziz et al. [29] who found value of 6.46 g/L in effluent generated from the three-phase extraction system. The amount of 1.34 g/L detected in the present work was comparable to 1.8 g/L reported by Dammak [18] and within the range 0.03–6.13 g/L reported by Davies et al. [30]. The variability in total phenol concentration may be attributed to several factors, such as the olive cultivars and their degree of ripening. Moreover, the agronomic conditions and treatments applied to extract the olive oil and to treat the OMW, may also significantly impact the quantitative and qualitative phenolic content and solid residues of OMW [5,31]. The major compounds detected in OMW are HT and TY, with HT known for its high antioxidant activity [32]. Dermeche et al. [5] in their review reported that in OMW, HT is found in the range of 20–1224 mg/L, while TY is between 145–208 mg/L. Regarding OLE, it has not been detected in OMW deriving from late-harvest olives [31], and its content has been described as highly variable [11,29,33].

The solid phase recovery of polyphenols from OMW has been successfully proposed using adsorbent resins, particularly with the S-DVB-based resins [14,15,34]. In the present work, in order to optimize the process, different resins, including the SP207, never employed before in the recovery of phenolic compounds from OMW, were tested. All the tested resins were capable of successfully adsorbing polyphenols [34,35], with the SP207 resin showing a high and selective affinity for OMW polyphenols. This non-polar resin, generally used for the recovery of phenolic compounds from citrus, apple, and winery by-products and wastes [36,37,38], showed the best efficiency in terms of total phenols concentration and selective recovery of the main OMW bioactive compounds. This type of approach to the recovery of bioactive compounds from OMW is desirable for the scale-up from laboratory to industrial production thanks to the simple and reproducible operating conditions and to the low costs of the process.

The enzymatic activity, as expected, highlighted the absolute supremacy of the *A. niger* strain, confirming its successful employment at an industrial level to produce purified enzymes [11]. The hyphal mode of fungal growth and its robustness to a wide range of pH and water activity make fungi extremely efficient in the bioconversion of solid substrates [39]. In fact, live *A. niger* culture showed an enzymatic activity up to 45-fold higher than the commercial enzyme. The *W. anomalus* and *L. plantarum* tested strains gave interesting results. In particular, *W. anomalus* showed a beta-glucosidase activity statistically equal to that of the commercial enzyme, and an esterase value higher than that of the commercial enzyme. Despite its potential to produce enzymes of technological importance, *W. anomalus* has been scarcely explored in food and byproduct applications.

The interest in microbial enzymes for biotechnology applications has grown above all in the bioconversion of industrial by-products [40]. The enzymatic test assessed in the present work highlighted the importance of testing microbial cultures already known for their technological or probiotic properties. A considerable amount of research has been conducted to determine the enzymatic properties of LAB [41], while other studies reported a strong β-glucosidase activity for *W. anomalus* yeast [42,43,44,45,46]. The bioconversion test showed that *W. anomalus* and *L. plantarum* were able to produce more HT than the commercial enzyme after 2 h testing. Moreover, the bioconversion test highlighted that microbial live cells were more effective in increasing the HT and TY content than their filtered broths, probably because the phenol extract, generating stressful conditions, could induce a stimulating effect on microbial metabolism. The statistically significant increase in TY (the TY recently highlighted and quantified to apply the EFSA health claim on olive oil polyphenols) obtained using the single pellet of both *W. anomalus* and *L. plantarum* cultures, is also valuable [47]. In the present work, the OLE value was detected as increased, but this may be due to a depolymerizing effect that microbial enzymes may have had on OMW polyphenols.

## 4. Materials and Methods

### 4.1. Raw Material Treatment

Fresh OMW was sampled in October 2018 from an olive oil producing plant of a factory located in Catania, Italy, which used a three-phase continuous oil extraction system. Samples were centrifuged at 10,000 rpm for 5 min at +4 °C, then filtered (Miracloth paper, Calbiochem, Canada) and stored at −20 °C until further analyses.

### 4.2. Extraction of Phenols from OMW

Four commercial adsorbent resins, Amberlite XAD-16 (Sigma Aldrich, Milan, Italy), Sepabeads SP-207 (Mitsubishi Chem. Co., Tokyo, Japan), Purosorb PAD900C (Purolite, Milan, Italy), and Hamilton C18 (Thermo Fischer Scientific, San Jose, CA, USA) were selected and tested. XAD-16 and SP-207 are styrene-divinylbenzene (S-DVB) copolymers; PAD900C is polydivinylbenzene polymer; and finally, C18 having Poly(S-DVB) matrix was used as analytical reference.

Before their employment, the adsorbent resins were pretreated with 95% ethanol (food grade, Alcoolita, Italy), washed with water (HPLC grade, Carlo Erba, Italy), and then oven-dried at 70 °C up to constant weight. All adsorption experiments were conducted with determined quantities of activated dried resins. Then, 1 bed volume (BV) of each dry resin (about 20 mL) was loaded on a glass preparative column (length, 30 cm; i.d., 0.5 cm) connected with a peristaltic pump. After rinsing the resins with water, the OMW, pretreated as described in Section 4.1, was passed on resins until their saturation. Loading was stopped when the outflow OMW reached the same absorbance values and color of the loaded OMW. Before the desorption phase, each saturated resin was washed with 4 BV of water to remove water-soluble compounds. The adsorbed phenolic fraction of each resin was then recovered with 2 BV of a 95% ethanol/water solution (60:40, *v/v*). The collected desorbed fractions were finally concentrated after vacuum distillation of ethanol at 40 °C by using a rotary evaporator (Rotavapor RE111, Büchi, Cornaredo, Italy).

### 4.3. Physicochemical Characterisation

The pH value of OMW samples was measured using a Mettler DL25 pH meter (Mettler-Toledo International Inc., Columbus, OH, USA). Dry weight was determined by weighing the samples before and after drying in an oven at 105 °C up to constant weight.

The total phenolic content of OMW and extracts was determined according to the Folin–Ciocalteu’s (FC) colorimetric method. The OMW samples were mixed with 5 mL of FC commercial reagent (Labochimica, Italy) diluted with water 1:10, *v/v*, and 4 mL of a 7.5% sodium carbonate solution. After stirring for 2 h at room temperature away from light, the absorbance of the blue solution was measured spectrophotometrically at 765 nm (Cary 100 Scan UV-Visibile, Agilent, CA, USA). The total phenolic content was expressed as mg of gallic acid equivalents (GAE)/L of the sample.

### 4.4. HPLC Analysis

HPLC analyses of phenol fraction of OMW and extracts were obtained by directly injecting the filtered samples (0.45 μm Millipore filters) in the chromatographic HPLC system. The system consisted of a liquid chromatography Waters Alliance 2695 HPLC equipped with a Waters 996 photodiode array detector (PDA) set at 280 nm and with Waters Empower software (Waters Corporation, MA, USA). The column was a Luna C18 (250 mm × 4.6 mm i.d., 5 μm, 100 Å; Phenomenex, Torrence, CA, USA) maintained in an oven at 40 °C. Chromatographic separation was achieved by elution gradient using an initial composition of 95% of A solution (water acidified with 2% acetic acid) and 5% of B solution (methanol). Solvents were HPLC grade (Merck KGaA, Darmstadt, Germany). The B solution increased to 30% in 15 min and to 70% in 25 min and then, after 2 min in isocratic, the mobile phase was set at the initial conditions for 8 min. A flow of 1 mL/min was used [48]. The internal standard (I.S.) of 5 mM pure gallic acid (Fluka, Switzerland) was used to quantify the phenolic compounds. The identification of phenolic compounds was obtained by comparing retention time with pure tyrosol, oleuropein, and hydroxytyrosol standards (Extrasynthese, Genay, France). All the analyses were carried out in triplicate for each sample.

### 4.5. Microorganisms

*Lactiplantibacillus plantarum* DSM 20205, *Saccharomyces cerevisiae* DSM 1333, and *Aspergillus niger* DSM 2466, were all purchased from Leibniz-Institute DSMZ, German collection. Moreover, *Wickerhamomyces anomalus* F6.05, a strain isolated from brine samples of naturally fermented table olives, was from the Culture Collection of the Department of Agricultural, Food, and Environment (Di3A), University of Catania, Italy. All the used cultures were maintained as stock solution in 20% (*v/v*) glycerol at −80 °C before use. *A. niger* stock suspension was obtained by growing the strain on Potato Dextrose Agar (PDA, Oxoid, UK) at 30 °C for 5 days and by harvesting the spores with 5 mL Tween 80, 2% (*v/v*) in sterile water. The spore solution was stored in 20% (*v/v*) glycerol at –20 °C before use.

### 4.6. Enzyme Production Test

The *L. plantarum* strain was cultured at 32 °C for 24 h in De Man, Rogosa, and Sharpe broth (MRS broth, Oxoid, Milan, Italy) while *S. cerevisiae* and *W. anomalus* strains were cultured at 30 °C for 24 h in Yeast Malt broth (YM broth, Merk, Italy). A cell density of 10^8^ CFU/mL of each microorganism was used to inoculate test tubes containing media without a carbon source, MRS broth without Dextrose (Likson, Italy) for *L. plantarum*, and yeast nitrogen base (Likson, Italy) for *S. cerevisiae* and *W. anomalus* strains. After an overnight culture at the same temperature shown above, the samples were filtered with 0.45 μm paper filters (Minisart, Sartorius Stedim, France) to collect the filtrate containing the extracellular enzymes for subsequent enzymatic tests.

The *A. niger* strain was used as a positive control by using the method reported by Hamza et al. [11]. Flasks containing 100 mL of yeast nitrogen base (YNB, Likson, Italy) with 6.7 g of wheat bran (Ki, Italy) as carbon source were inoculated at 10^7^ spores/mL concentration. The flasks were left in agitation at 120 rpm for 10 days at 25 °C. The culture broth was filtered through a muslin cloth to collect the filtrate containing the extracellular enzymes.

### 4.7. Beta-Glucosidase Activity

Beta-glucosidase activity was measured determining the quantity of p-nitrophenol released from the p-nitrophenyl-beta-D-glucopyranoside (p-NPG) by the enzymatic activity of the tested strains. The activity was determined according to the method of Khoufi et al. [16], slightly modified. For the enzymatic assay, the incubation mixture contained 0.9 mL of 5 mM p-NPG (Sigma-Aldrich, Milan, Italy) in 50 mM citrate buffer (pH 4.8), and 0.1 mL of centrifuged broth from each overnight culture. The reaction was maintained at 50 °C for 2 h and stopped by the addition of 2 mL of Na_2_CO_3_ buffer (1.0 M). The amount of p-nitrophenol (p-NP) released was spectrophotometrically determined at 400 nm and quantified by using p-NP (Glentham, Life Science, UK) to obtain a standard curve. One unit (IU) of enzyme activity was defined as the amount of enzyme that produced 1 μmol of p-NP/min under the same conditions. The enzymatic results were expressed as IU/mL of sample by means of the following equation: IU/mL = Q/T × C/V, where Q is the quantity (µmole) of p-NP generated at the time T (min), V is the volume (mL) of the sample, and C is the total reaction volume (mL).

### 4.8. Esterase Activity

Esterase activity was determined according to the method of Mackness et al. [49]. The reaction mixture contained 50 μL of centrifuged broth from each overnight culture, 50 μL of 150 mM p-nitrophenyl-acetate (p-NPA, Acros Organics, Fair Lawn, NJ, USA) in ethanol, and 2.9 mL of Tris-HCl buffer (pH 7.5). The solution was incubated at 25 °C for 4 min. The amount of p-NP released was spectrophotometrically determined at 400 nm and quantified by using p-NP to obtain a standard curve. The enzymatic results were expressed as described in Section 4.7.

### 4.9. Bioconversion of Phenolic Compounds

The OMW extract obtained by the SP207 resin was used for the proposed enzymatic hydrolysis aimed at hydroxytyrosol increase. The procedure described in the enzyme production test above mentioned (Section 4.6) was repeated. In this case, only *L. plantarum* and *W. anomalus*, the two strains that showed the highest enzymatic activity, were tested. The two overnight cultures were centrifuged to collect separately the pellets and supernatants. The pellets were centrifuged twice at 5000 rpm for 5 min at +4 °C in sterile water and standardized at the same cell density of 10^8^ CFU/mL in sterile water.

Bioconversion reactions were assessed according to Hamza et al. [11], with modifications. The reactions were conducted in Erlenmeyer flasks by adding 5 mL of cultured samples (both pellet and filtered supernatant were tested separately) and 15 mL of OMW extract (diluted 1:10 in water). The enzymatic reaction was performed at 50 °C for 6 h under static conditions. Samples were collected every hour up to 6 h, and then filtered for subsequent analyses for phenols by HPLC.

Moreover, the enzymatic activity of all the samples was compared to the activity of Lallzyme Beta (Lallemand, Blagnac, France) a commercial enzyme produced by *A. niger*, with β-glucosidase and polygalacturonase activity. The enzyme was used as a positive control (5 mL) at a dose of 5 mg/100 mL sterile water, in accordance with the manufacturer’s instructions. The negative control was incubated with 5 mL of sterile water.

### 4.10. Statistical Analysis

All analyses carried out were performed in triplicate. SPSS software (version 21.0, IBM Statistics, Armonk, NY, USA) was used for data processing. Statistical analysis of the results was performed using one-way analysis of variance (ANOVA), and Tukey’s HSD post hoc test for means separation at a significance level of *p* ≤ 0.05.

## 5. Conclusions

The effects of different adsorbent resins were tested to obtain an OMW extract rich in bioactive compounds. The OMW extract obtained from the SP207 resin showed the best efficiency in selective recovery of the main OMW bioactive compounds. The beta-glucosidase and esterase activity of different microorganisms, already known for their technological or probiotic properties, were investigated and compared to those of a commercial enzyme and an *A. niger* strain. Remarkably, the results demonstrated that the *W. anomalus* and *L. plantarum* tested strains were the most effective in increasing HT and TY, especially when they were used as pellets in contact with the OMW extract. The results of the present study are promising and suggest the possibility to further explore liquid or solid formulations for antioxidant/nutraceutical supplements containing health promoting compounds and microorganisms.

## Figures and Tables

**Table 1:** Chemical parameters of olive mill wastewater (OMW) after pretreatment (centrifugation and filtration).

Parameters	Means ± SD
pH	4.80 ± 0.06
Dry matter (g/100 mL)	6.24 ± 0.02
Hydroxytyrosol (HT) (mg/L)	218.29 ± 4.94
Tyrosol (TY) (mg/L)	67.24 ± 5.57
Oleuropein (OLE) (mg/L)	207.49 ± 56.37
Total phenols (mg/L)	1343.26 ± 0.54

**Table 2:** Phenols detected in OMW extracts obtained with the different tested resins.

Adsorbent Resin	Hydroxytyrosol (HT)	Tyrosol (TY)	Oleuropein (OLE)	Total Phenols
SP207	3239.7 ± 57.34 a	2016.8 ± 49.36 a	11,336.6 ± 199.65 a	16,447.9 ± 16.31 a
XAD16	1381.7 ± 66.45 b	1000.3 ± 120.75 b	10,047.8 ± 120.29 ab	13,144.6 ± 21.75 b
PAD900C	294.3 ± 2.67 c	90.0 ± 10.59 c	4443.8 ± 176.32 c	4880.3 ± 10.87 d
C18	191.1 ± 16.59 c	50.4 ± 10.34 c	8092.3 ± 218.15 b	8763.6 ± 43.50 c

Data are expressed as mg/L of means ± standard deviations. Different letters indicate statistical differences within the same column (Significance at *p* < 0.001).

**Table 3:** Beta-glucosidase and esterase activities detected for the commercial enzyme and the different microorganisms tested under their specific growth culture conditions.

Samples	Beta-Glucosidase	Esterase
Lallzyme beta enzyme	6979.9 ± 3.22 b	316,713.9 ± 1032.48 c
*W. anomalus*	7066.4 ± 5.36 b	405,417.6 ± 516.24 b
*L. plantarum*	2387.6 ± 3.22 c	316,348.9 ± 1540.62 c
*S. cerevisiae*	1437.9 ± 78.31 d	399,577.0 ± 1548.72 b
*A. niger*	314,313.8 ± 107.28 a	4,506,229.2 ± 4646.15 a

Data are expressed as IU/mL of broth sample (means ± standard deviations). Different letters indicate statistical differences within the same column (Significance at *p* < 0.001).

## Supplementary Materials

The following are available online. Table S1: Kinetics of bioconversion of single phenols analyzed through HPLC.

## Sample Availability

Samples of the OMW extracts as obtained and described in this paper are available from the authors.
