# Supplementary material for: Microbial Application to Improve Olive Mill Wastewater Phenolic Extracts
Source: Molecules. 2021 Mar 30;26(7):1944. doi: 10.3390/molecules26071944 (PMC8036537; doi:10.3390/molecules26071944)
Supplement: Supplementary file 1 [file molecules-26-01944-s001.pdf]

Supplementary material

Table S1. Kinetics of bioconversion of single phenols analyzed through HPLC.

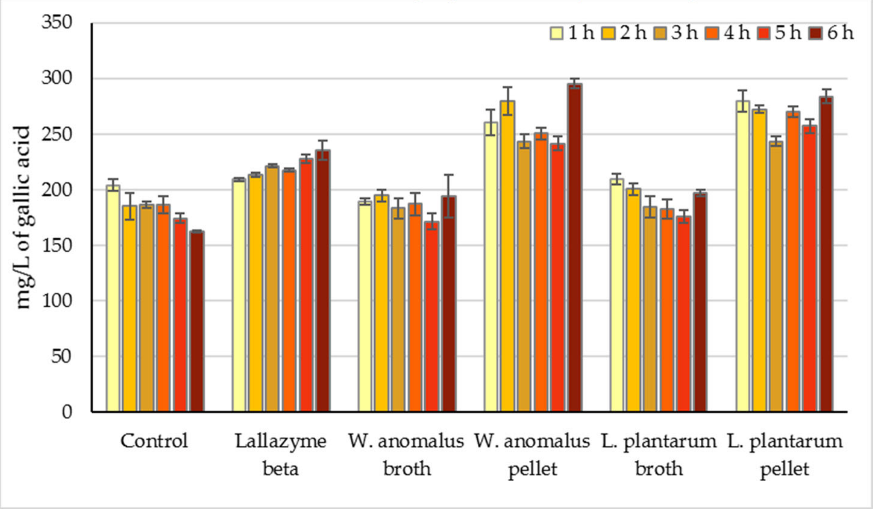

A) Hydroxytyrosol

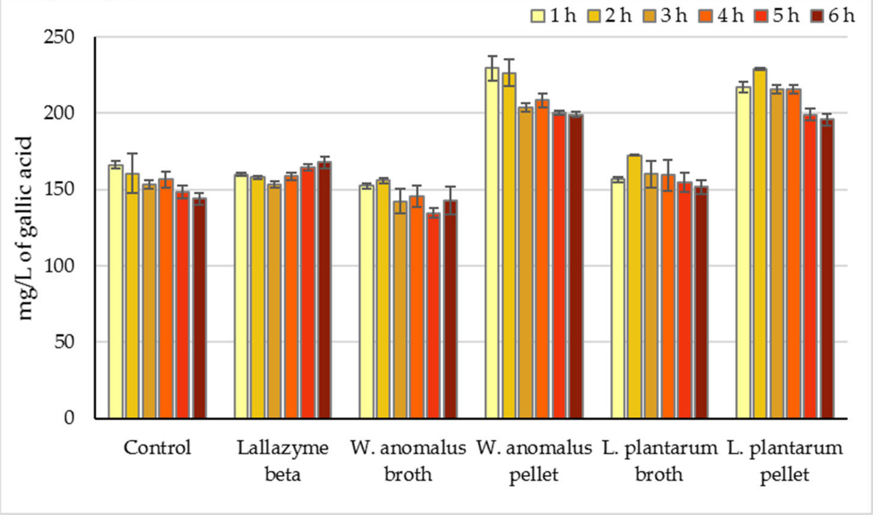

B) Tyrosol

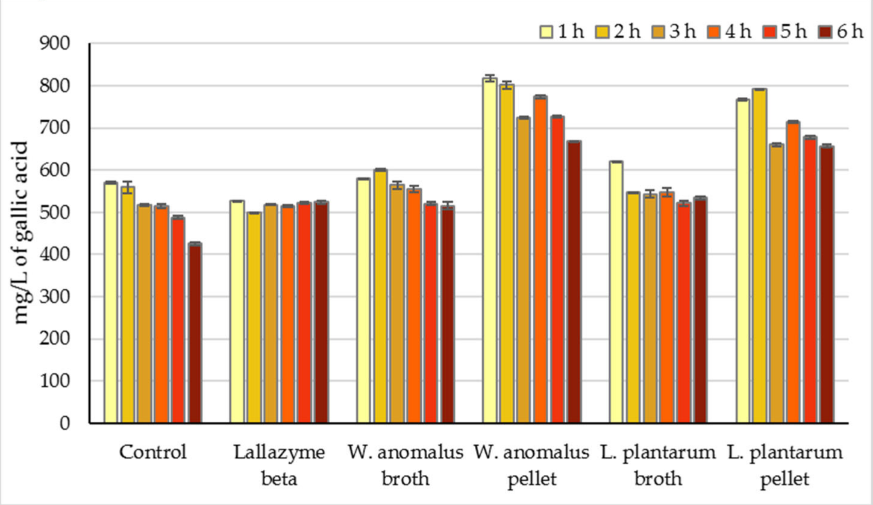

C) Oleuropein
